# Primacy and recency effects as indices of the focus of attention

**DOI:** 10.3389/fnhum.2014.00006

**Published:** 2014-01-24

**Authors:** Alexandra B. Morrison, Andrew R. A. Conway, Jason M. Chein

**Affiliations:** ^1^Department of Psychology, Temple University, PhiladelphiaPA, USA; ^2^Department of Psychology, Princeton University, PrincetonNJ, USA

**Keywords:** working memory, focus of attention, primacy and recency effects, phonological rehearsal, medial temporal lobe

## Abstract

Ongoing debate surrounds the capacity and characteristics of the focus of attention. The present study investigates whether a pattern of larger recency effects and smaller primacy effects reported in previous working memory studies is specific to task conditions used in those studies, or generalizes across manipulations of task-demand. Two experiments varied task-demands by requiring participants to remember lists of letters and to then respond to a subsequent two-item probe by indicating either the item that was presented later in the list (judgment of recency) or the item was presented earlier (judgment of primacy). Analyses tested the prediction that a WM task emphasizing later items in a list (judgment of recency) would encourage exaggerated recency effects and attenuated primacy effects, while a task emphasizing earlier items (judgment of primacy) would encourage exaggerated primacy effects and attenuated recency effects. Behavioral results from two experiments confirmed this prediction. In contrast to past studies, fMRI contrasts revealed no brain regions where activity was significantly altered by the presence of recency items in the probe, for either task condition. However, presence of the primacy item in the probe significantly influenced activity in frontal lobe brain regions linked to active maintenance, but the location and direction of activation changes varied as a function of task instructions. In sum, two experiments demonstrate that the behavioral and neural signatures of WM, specifically related to primacy and recency effects, are dependent on task-demands. Findings are discussed as they inform models of the structure and capacity of WM.

## Introduction

A wide variety of research suggests strict limits on the mind's ability to maintain and manipulate information over the short term—an ability often referred to as working memory (WM; e.g., Luck and Vogel, 1997; Cowan, 2001; McElree, 2006). While researchers generally agree on the existence of such capacity limits, ongoing debate surrounds the precise structure and function of human memory, including the specific properties and capacity limits of WM (Miyake and Shah, 1999; Jonides et al., 2008). The present paper explores one explanation for divergent conclusions about the architecture and capacity of WM, and concentrates on the contributions of the “focus of attention” (FOA, or the most immediate state of WM) to WM capacity (Cowan, 1995). Using both behavioral and neuroimaging (fMRI) methods, we test the hypothesis that subtle features of the tasks used to probe WM function (e.g., task instructions and response requirements) can lead to important performance differences (e.g., which item in a list is remembered the quickest), fundamental changes in the pattern of brain activity evoked by the WM task, and ultimately, to different conclusions about the FOA and its involvement in WM.

A set of recent fMRI studies inform our approach (Talmi et al., 2005; Nee and Jonides, 2008, 2011; Öztekin et al., 2009, 2010). Each of these studies set out to test theoretical claims about WM by comparing the brain activation patterns associated with retrieval of items from different serial positions in a list, an idea based on earlier behavioral research (Postman and Phillips, 1965; Glanzer and Cunitz, 1966). Memory for lists of items almost always yields evidence of primacy and recency effects—elevated memory for the earliest and latest items compared to middle items (Postman and Phillips, 1965; Glanzer and Cunitz, 1966). By some accounts, primacy and recency effects reflect separate memory stores (Talmi et al., 2005) or states (Nee and Jonides, 2011), and in general the neuroimaging findings have been consistent with this interpretation.

The earliest imaging study to investigate serial position effects showed that recognition of the final two items in a 12 item list was accompanied by higher inferior parietal activation, while recognition of the two earliest items was accompanied by increased activation of the left medial temporal lobe and frontal areas including the bilateral inferior frontal gyri (BA, 45 and 47) and bilateral middle frontal gyri (BA 8 and 9) (Talmi et al., 2005). Recognition of the most recent items also was also faster and more accurate than recognition of the first two items. These neural and behavioral differences were presented as support for distinct memory stores associated with early and late items.

Other neuroimaging investigations using serial position comparisons sought to test more directly whether brain activity associated with retrieval of the final list item might be different from that associated with retrieval of earlier list items. The motive for testing this specific contrast came from behavioral evidence indicating a selective retrieval advantage for the final item in a list (McElree and Dosher, 1989, 1993), which some investigators have interpreted as support for WM theories positing that the most recently encountered item is maintained in an especially accessible state, a single item FOA (McElree, 2006).

Thus, comparison of trials involving recognition of the final item with trials involving other subsets of to-be-remembered items might reveal the FOA (Talmi et al., 2005; Nee and Jonides, 2008, 2011; Öztekin et al., 2009, 2010). Consistent with this expectation, Nee and Jonides (2008) administered a three item word recognition task and found that, when compared to the two earlier words, the final item in a list was recognized faster and accompanied by more activation in the inferior temporal cortex and less activation in the medial temporal lobes. Similar results were obtained by Öztekin et al. (2010), who administered a 10 word recognition task and found higher accuracy, faster reaction times, and less hippocampal activation for the most recent item when compared to all other items.

A more recent study from Nee and Jonides (2011) was designed to test an even more nuanced claim derived from Oberauer's three tiered model of WM (Oberauer, 2002 and see Cowan, 1995). This model assumes not only a single item FOA, but also an expanded set of items held within a “region of direct access” in short-term memory. These items are associated with an intermediate state of accessibility. In the study, brain activity associated with recognition of items in each of three separate positions in a six item list (assumed to be representative of the single-item FOA, the multi-item region of direct access, and activated long-term memory) was examined. The imaging evidence was consistent with the three state account. Most importantly, like the previous studies exploring serial position effects at retrieval, the study found that recognition of the final item was faster, and accompanied by less medial temporal lobe (MTL) activation and more inferior temporal and left inferior parietal activation than recognition of items from elsewhere in the list.

A final study, which was used as a model for the present study, compared retrieval of items in different serial positions in a judgment of recency (JOR) task (Öztekin et al., 2009). Here, subjects were presented with five letter lists, with letters presented one at a time. After a short mask, a probe appeared containing two of the items, and subjects were asked which item came later in the list. In order to measure performance by serial position, trials were grouped by the serial position of the correct probe (Muter, 1979; Hacker, 1980; Hockley, 1984; McElree and Dosher, 1993). When the correct judgment involved recognition of the item that had been presented in the final serial position, performance was faster and more accurate, and fMRI contrasts revealed reduced left hippocampal and left inferior frontal (BA 45) activation, relative to trials for which the more recent of the probed items had not been in the final serial position.

The five imaging studies just reviewed provide evidence regarding the neural substrates of the behavioral recency effect. Moreover, by showing that retrieval of the final list item relies on a different set of neural processes (e.g., less hippocampal involvement), these studies seem to provide convergent support for theoretical accounts attributing a special status to a single item held within the FOA. It is noteworthy; however, that the studies comprising this work all involved highly similar testing procedures. This is important because it is known that the magnitude of the behavioral recency effect varies substantially as a function of the particular task used to probe memory (Oberauer, 2003). Item recognition and JOR tasks (especially with verbal items) tend to produce relatively exaggerated recency effects and attenuated primacy effects, in comparison to other memory tasks (McElree and Dosher, 1989). Meanwhile, other tasks, such as immediate serial recall, tend to produce exaggerated primacy effects and attenuated recency effects (Jahnke, 1963, 1965).

Accordingly, the present work examines whether the findings obtained from earlier imaging investigations of the serial position curve might be contingent on the specific task demands present in those studies, and not reflective of a general feature of item representation in the FOA. Toward this end, we deployed a JOR task closely resembling that used in Öztekin et al. (2009), but in addition, introduce a novel “judgment of primacy” task. In the judgment of primacy (JOP) task, subjects were asked to identify which of two test items had been presented *earlier* in the preceding list of items. Thus, the JOR and JOP task trials were identical except for the requirement of choosing the later (JOR) or earlier (JOP) item of the two items in the retrieval probe.

By exploring the behavioral and neural correlates of JOP performance, and comparing them to the correlates of JOR performance, we hoped to achieve two objectives. First, we tested the prediction that representation in the FOA is not invariably tied to the last item in a list, but rather, that the FOA can be allocated flexibly according to task demands. Specifically, we anticipated that JOR instructions would place a premium on retention of later items in the list, while JOP instructions would encourage participants to emphasize retention of earlier items within the FOA. Behaviorally, we anticipated that this reallocation would impact the magnitude of the recency (stronger in JOR) and primacy (stronger in JOP) effects. In the brain, we anticipated that reallocation of the FOA would alter the pattern of activity produced by recognition of items from different serial positions. We predict the pattern of less hippocampal activity for retrieval of recency items relative to other items will be seen in JOR but will not generalize to JOP. Contrary to our predictions, evidence in favor of a single item FOA tied to the final item would emerge if both JOR and JOP revealed similar neural and behavioral correlates associated with retrieval of the final item.

A second motive for including JOP was to examine the relationship between primacy effects in WM and representation within the FOA. Although behavioral primacy effects were evident in prior studies, an emphasis on the relationship between recency and the FOA caused the neural correlates of primacy to be essentially overlooked. For example, Nee and Jonides (2011) dissociated three different groupings for the six items in their lists (the second and third item, the fourth and fifth items, and the sixth item). However, the first item in the list “was excluded in neural analyses due to ambiguity surrounding the primacy effect” (Nee and Jonides, 2011, p. 1541). Indeed, there is at present little clarity regarding the memory or attentional state of the primacy item. Thus, a further aim of the present work was elucidation of characteristics of primacy effects and also their relationship to the status of representation in WM.

A final objective in the current work was to investigate the role of the hippocampus and surrounding MTL in WM. Historically, the MTL has not been implicated in WM, in large part because damage to the MTL is associated with profound long-term memory deficits but typically leaves short-term memory intact (Squire and Schacter, 2002). Most neuroimaging studies of short-term and WM have, consistent with earlier neuropsychological findings, failed to elicit activation of the MTL (Wager and Smith, 2003; Owen et al., 2005). Recent studies probing the hippocampus in the retrieval period of JOR and item recognition tasks (Talmi et al., 2005; Nee and Jonides, 2008, 2011; Öztekin et al., 2009, 2010), however, demonstrated that retrieval of the final item (which is putatively held within the FOA) is accompanied by reduced activation in the hippocampus. Through serial position contrasts examining a variety of items on the serial position curve (i.e., early and middle items in addition to the final item), the present study aimed to further elucidate the role of MTL activity in retrieval of items in a WM task.

In sum, there are competing accounts of the structure of WM. While recent papers argue that a single item recency effect is indicative of a single item FOA, this phenomenon has only been examined for a narrow set of tasks. Here, the generalizability of recency effects and their neural correlates is revisited in order to inform the use of the recency effect as a marker of the FOA. Of primary interest to the current paper are the following questions: Are the magnitudes of primacy and recency effects dependent on task-demands? And, do primacy and recency effects have consistent neural signatures across tasks? These questions are probed in two parallel experiments, one behavioral and one using fMRI, and the results are discussed as they inform the structure of WM and conceptions of the relationship between WM and the FOA.

## Experiment 1

### Methods

#### Subjects

The sample consisted of 20 undergraduate students (14 female; *M* = 20.35 years old, *SD* = 1.50 years). All subjects received course credit for participation.

#### Tasks

**Figure 1:**
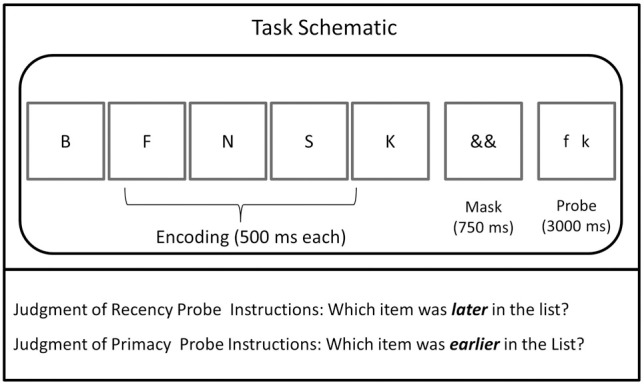
**Schematic of the tasks from Experiments 1 and 2**. The JOR and JOP tasks employed identical timing and stimuli but differed in whether participants were instructions to report the later or earlier of the two items in the probe.

There were two task types, JOR and JOP. Each task required memory for letters (selected from a pool of 16 consonants: B, C, F, H, J, K, L, M, N, Q, R, S, V, W, X, and Z), and participants completed 96 trials per task. Each trial was initiated by the subject's key press and then followed the same sequence: 500 ms fixation, five items presented sequentially for 500 ms each, 750 ms mask, and a 3-second probe, which included two items from the trial (Figure 1). Trial presentation was identical across the JOR and JOP tasks, except for the requirement that subjects report the earlier (JOP) or later item (JOR) in the probe. The probe remained on the screen for 3 s, regardless of whether the subject responded.

Trial sequences were constructed prior to the experiment in order to control the serial position of the correct and incorrect probes. The two items contained in each probe were counterbalanced so that in half of trials the correct answer was on presented on the right side of the screen and half the trials it was on the left side of the screen. Tasks were constructed so that each of the four possible correct serial positions was probed as the correct response 24 times, resulting in 96 trials per task, and the serial position of the incorrect probe was also balanced. Trials were blocked by task so that subjects would not be required to rapidly switch task-demands between trials. Task order was counterbalanced across subjects. Within each task block of 96 trials, the order of trials probing different serial positions was randomized, as was the identity of the letters presented in each serial position. In an attempt to encourage verbal phonological coding, and to discourage simple perceptual matching of the encoded and probed items, capital letters were used for item presentation while lowercase letters were used for retrieval probes.

#### Procedure

In each task, subjects completed 5 practice trials, followed by 96 actual trials. Each task block lasted roughly 15 min (each trial was 6.75 s, and the inter-trial intervals were subject-paced), and subjects were allowed to pause between blocks.

#### Data analysis

All significance tests were conducted from the perspective of null hypothesis significance testing (NHST) and were non-directional with alpha = 0.05. To supplement the NHSTs, effect size estimates were calculated using partial eta-squared. Behavioral measures included both accuracy and reaction times, but reaction time on correct trials was the main outcome of interest for examining serial position dynamics. Serial position analyses proceeded with two separate methodologies for defining primacy and recency trials and calculating primacy and recency effects.

***Serial position analysis, method 1: primacy and recency assigned according to the position of the correct response.*** The first method of analysis was based on Öztekin et al. (2009) and earlier investigations of JOR (see McElree, 2006), and involved averaging of trials according to the serial position of the correct response. Here, primacy and recency trials were defined by whether the earliest or latest possible correct response was included in the probe. In JOR, primacy trials were trials where the correct response was item 2 (1–2 probes), middle trials were trials where the correct response was item 3 or 4 (1–3, 1–4, 2–3, 2–4, and 3–4 probes), and recency trials were trials where the correct response was item 5 (1–5, 2–5, 3–5, and 4–5 probes). In JOP, primacy trials were trials where the correct response was item 1 (1–2, 1–3, 1–4, and 1–5 probes), middle trials were trials where the correct response was item 2 or 3 (2–3, 2–4, 2–5, 3–4, and 3–5 probes), and recency trials were trials where the correct response was item 4 (4–5 probes).

In both JOR and JOP, separately, primacy effects were calculated as a percent change with the following procedure: (1) reaction times on middle trials were averaged; (2) the average of the reaction times for primacy trials was subtracted from this average; (3) the resulting difference was divided by the average of the middle items; and (4) the result was multiplied by 100. Recency effects were calculated with the following, analogous, procedure: (1) reaction times for middle trials were averaged; (2) the average of the reaction times for recency trials was subtracted from this average; (3) the result was divided by the average of the middle trials, and (4) the result was multiplied by 100. The procedure produces an index of the advantage (or decrement) for the retrieval of the primacy/recency item in proportion to the reaction times of other items in the list.

***Serial position analysis, method 2: primacy and recency assigned according to whether the first or last items are included in the probe.*** A second method of condition assignment involved consideration of the serial position of both the correct and incorrect probe. Here, primacy and recency were defined according to whether the primacy item (1st) or recency item (5th) was contained in the probe (as either the correct or incorrect response). This method provides a more direct comparison of primacy and recency across the two task types and takes advantage of the fact that the probes comprise the same exact serial positions in both tasks. Within each task, trials were averaged into four types: (1) primacy trials for which probes included the first item from the list, but not the last (1–2, 1–3, and 1–4 trials); (2) recency trials for which probes included the last item from the list but not the first (5–4, 5–3, and 5–2 trials); (3) middle trials for which the probe contained neither the first not the last item (2–3, 2–4, and 3–4 trials), and (4) 1–5 trials that contained both the primacy and recency items. In JOP and JOP, separately, the magnitude of the primacy effect was calculated by subtracting the reaction time on correct primacy trials from the reaction time on correct middle trials, dividing this value by the reaction time on correct middle trials, and then multiplying the product by 100. The magnitude of the recency effect was calculated by subtracting the reaction time on correct recency trials from the reaction time on correct middle trials, dividing this value by the reaction time on correct middle trials, and then multiplying the product by 100.

### Results

Accuracy for both task-types was well above chance (JOR: *M* = 0.85, *SD* = 0.07; JOP: *M* = 0.86, *SD* = 0.07) and average reaction times in milliseconds (ms) for correct trials were as follows (JOR: *M* = 1560.51, *SD* = 206.33; JOP: *M* = 1432.46, *SD* = 209.52).

#### Method 1. primacy and recency assigned according to the position of the correct response

**Figure 2:**
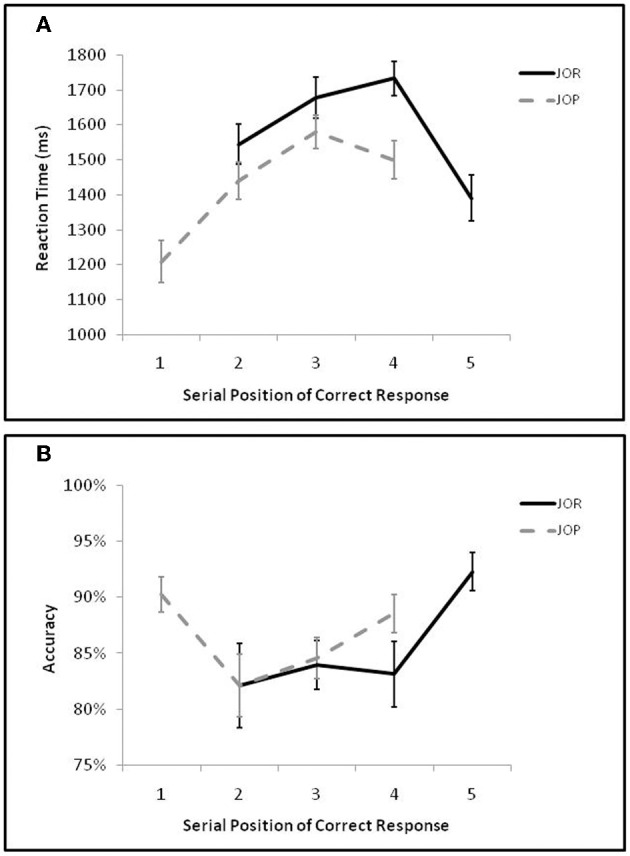
**(A)** Average reaction time on correct trials as a function of the serial position of the correct item in the JOR and JOP tasks. **(B)** Average accuracy as a function of the serial position of the correct item in the JOR and JOP tasks.

As in Öztekin et al. (2009) initial analyses compared performance as a function of the serial position of the correct response (Figure 2). A 2 by 4 repeated measures ANOVA examining the interaction of task-demand (JOR, JOP) and serial position of the correct response (JOR: 2, 3, 4, 5; JOP: 1, 2, 3, 4) on task accuracy revealed no main effect of task [*F*_(1, 19)_ = 0.53, *p* = 0.48, η^2^_*p*_ = 0.03] but a main effect of serial position [*F*_(3, 57)_ = 4.38, *p* = 0.019, η^2^_*p*_ = 0.44], and a marginal interaction of task and serial position [*F*_(3, 57)_ = 3.01, *p* = 0.059, η^2^_*p*_ = 0.35]. The main effect of position indicates the presence of a recency effect (JOR 5 and JOP 4 trials were more accurate than JOR 3, JOR 4, JOP 2, and JOP 3 trials, [*F*_(1, 19)_ = 14.36, *p* = 0.001, η^2^_*p*_ = 0.43]), and no accompanying primacy effect (JOR 2 and JOP 1 trials were equivalent to JOR 3, JOR 4, JOP 2 and JOP 3 trials [*F*_(1, 19)_ = 1.74, *p* = 0.20, η^2^_*p*_ = 0.08]).

Analysis of reaction times revealed a main effect of task where JOR was slower than JOP [*F*_(1, 19)_ = 25.51, *p* < 0.0005, η^2^_*p*_ = 0.57], a main effect of serial position [*F*_(3, 57)_ = 19.19, *p* < 0.0005, η^2^_*p*_ = 0.77], and an interaction between task and serial position [*F*_(3, 57)_ = 9.21, *p* = 0.001, η^2^_*p*_ = 0.62]. These analyses confirm performance differences associated with the serial position of the correct response, and the interaction of task and serial position found in the reaction time data suggests that serial position dynamics are not identical across tasks (see Figure 2). In fact, in JOR there was no significant primacy effect [e.g., primacy trials and middle trials were equivalent, *F*_(1, 19)_ = 0.75, *p* = 0.39, η^2^_*p*_ = 0.04] but a significant recency effect [recency trials were faster than middle trials, *F*_(1, 19)_ = 18.44, *p* < 0.0005, η^2^_*p*_ = 0.49], while in JOP there was a significant primacy effect [primacy trials were faster than middle trials, *F*_(1, 19)_ = 57.71, *p* = < 0.005, η^2^_*p*_ = 0.75], but no recency effect [recency trials and middle trials were equivalent, *F*_(1, 19)_ = 2.57, *p* = 0.13, η^2^_*p*_ = 0.12].

**Figure 3:**
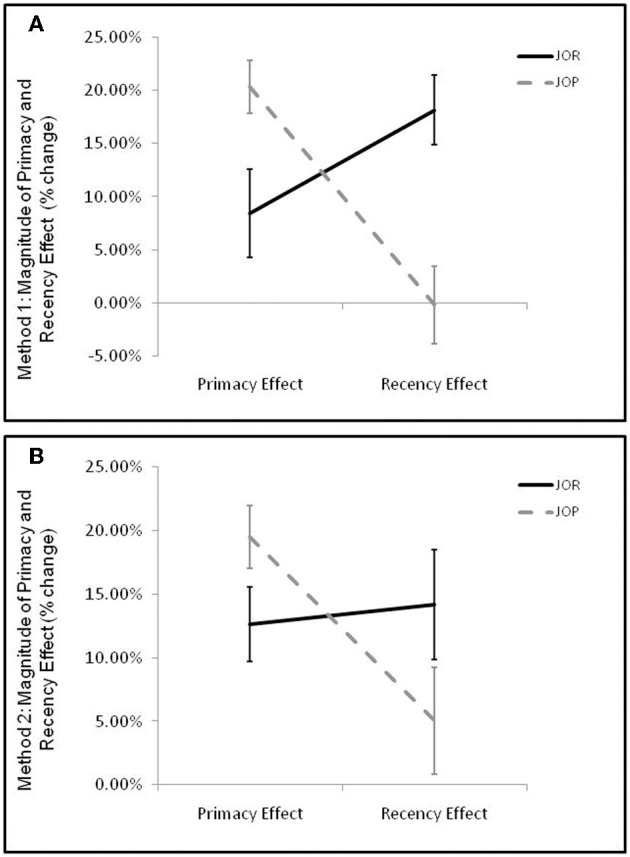
**Average magnitude of primacy and recency effects in JOR and JOP**. Error bars represent the standard error of the mean. Calculation of primacy and recency effects produces an index of the percent reaction time advantage for retrieval of the primacy/recency item when compared to retrieval of middle items. **(A)** Primacy and recency effects in JOR and JOP calculated using method 1. Method 1 divides trials by the serial position of the correct probe item, and primacy and recency trials were defined by whether the earliest or latest possible correct response was the correct answer in the probe. **(B)** Primacy and recency effects in JOR and JOP calculated using method 2. Here, trials are divided by considering both items in the probe, and primacy and recency item trials are trials that include the earliest (primacy) or latest (recency) item.

Crucial to the aims of the present paper was whether the presence and magnitude of primacy and recency effects varied across task-demands. Primacy and recency effects, (calculated using method 1) were entered into a 2 by 2 repeated measures ANOVA examining the interaction between task-demand (JOR, JOP) and effect-type (recency effect, primacy effect) with the size of the primacy or recency effect as the dependent variable. Analysis showed no main effect of task-demand [*F*_(1, 19)_ = 1.13, *p* = 0.302, η^2^_*p*_ = 0.06] or of serial position effect [*F*_(1, 19)_ = 2.10, *p* = 0.17, η^2^_*p*_ = 0.10], but a significant effect-type by task-demand interaction [*F*_(1, 19)_ = 26.64, *p* < 0.005, η^2^_*p*_ = 0.58]. As predicted above, this interaction was driven by a larger primacy effect in JOP than JOR [*t*_(19)_ = 2.76, *p* = 0.01], accompanied by a larger recency effect in JOR than JOP [*t*_(19)_ = 4.48, *p* < 0.005] (Figure 3A).

#### Method 2. primacy and recency assigned according to whether the first or last item was included in the probe

A 2 by 4 repeated measures ANOVA examined the interaction of task-demand (JOR, JOP) and trial type (primacy trials, 1–2, 1–3, 1–4; recency trials, 5–4. 5–3, 5–2; middle trials, 2–3, 2–4, 3–4; and 1–5 trials) on reaction time for correct trials. This ANOVA revealed a main effect of task where JOR was slower than JOP [*F*_(1, 19)_ = 23.82, *p* < 0.005, η^2^_*p*_ = 0.56], a main effect of trial type [*F*_(3, 57)_ = 32.08, *p* < 0.005, η^2^_*p*_ = 0.85], and a trial type by task interaction [*F*_(3, 57)_ = 5.03, *p* = 0.01, η^2^_*p*_ = 0.47].

A series of paired comparisons exploring the significant interaction revealed that in JOR there was a significant primacy effect [i.e., primacy trials were faster than middle trials, *t*_(19)_ = 4.58, *p* < 0.005], a significant recency effect [i.e., recency trials were faster than middle trials, *t*_(19)_ = 3.44, *p* < 0.005]. Moreover, trials containing both the primacy and recency items were faster than either primacy item alone trials [*t*_(19)_ = 4.14, *p* < 0.005], or recency item alone trials [*t*_(19)_ = 5.29, *p* < 0.005]. In JOP there was a highly significant primacy effect [i.e., primacy trials were significantly faster than middle trials, *t*_(19)_ = 7.96, *p* < 0.001], no significant recency effect [i.e., no difference in speed between recency trials and middle trials, *t*_(19)_ = 0.63, *p* = 0.53] and trials including both the primacy and recency items (1–5) were equivalent to primacy item trials [*t*_(19)_ = 1.05, *p* = 0.31].

The magnitude of the primacy and recency effects were calculated within each task, this time using method 2, and the results were entered into a 2 by 2 repeated measures ANOVA examining the interaction between task-demand (JOR, JOP) and effect-type (recency effect, primacy effect). There was no main effect of task [*F*_(1, 19)_ = 1.619, *p* = 0.22, η^2^_*p*_ = 0.08], but the recency effect was smaller than the primacy effect [*F*_(1, 19)_ = 4.95, *p* = 0.038, η^2^_*p*_ = 0.42], and there was again a task-demand by effect type interaction [*F*_(1, 19)_ = 13.49, *p* = 0.002, η^2^_*p*_ = 0.42]. As with method 1, paired comparisons revealed a stronger recency effect in JOR than JOP [*t*_(19)_ = 2.57, *p* = 0.02], and a stronger primacy effect in JOP than JOR [*t*_(19)_ = 2.88, *p* = 0.01] (Figure 3B).

### Discussion

In order to evaluate primacy and recency effects and their comparative size in different task contexts, primacy and recency effects were examined as a function of task-demand (JOR, JOP). Primacy and recency effects were calculated both according to whether the earliest or latest potential correct response was the correct response in the probe, and as a function of whether the earliest or most recent item was contained in the probe (either as the correct or incorrect response). While studies of verbal JOR tasks have reported a small primacy effect and larger recency effect (McElree and Dosher, 1993; Öztekin et al., 2009), this pattern did not generalize across task-demands. Instead, both methods of calculating primacy and recency effects revealed that the comparative size of these effects differed between JOR and JOP. Specifically, there was a larger primacy affect in JOP than JOR, and a larger recency effect in JOR than JOP. Importantly, this pattern of results demonstrates an impact of task-demand on the size of primacy and recency effects, even when the two tasks possess identical stimulus presentations and basic response requirements. Indeed, these tasks shared every feature except for the requirement to identify the earlier or later of the two items presented in the probe. Arguably, tasks differing in more extensive ways would also produce differential serial position effects (although not necessarily in a single direction or additive fashion).

This experiment demonstrated behavioral evidence that the size of primacy and recency effects are dependent upon task conditions, and this finding is important in that it informs discussion of alternative views of the inherent structure of states within WM. Still, Experiment 1 focuses on behavioral indices alone and leaves under-determined whether these retrieval advantages come about from the same or different cognitive and neural processes. For example, it might be the case that the recency effect could arises from one cognitive process (e.g., the FOA) while the primacy effect arises from another (e.g., phonological rehearsal), but alternatively, both the primacy and recency effects could be supported by the same mechanism (e.g., allocation of the FOA). Moreover, the prior fMRI literature explores the neural underpinnings of a recency effect, but does not examine primacy effects, or compare the neural markers of primacy and recency effects. Thus, in a second experiment, fMRI was used to explore the neural correlates of both recency and primacy effects, and to examine whether these neural correlates are stable across task (JOR, JOP).

## Experiment 2

### Methods

#### Subjects

Twenty-eight individuals (18 female; *M* = 21.5 years old, *SD* = 2.8) participated in this experiment. Each subject was paid $30 for the 90-min session.

#### Experimental tasks

JOR and JOP tasks identical to the ones from Experiment 1 were used (Figure 1). Subjects completed the same 96 trials of JOR and 96 trials of JOP constructed for the behavioral experiment. However, unlike in the behavioral experiment, the inter-trial interval (ITI) was experimenter-paced, and varied between the trials (like Öztekin et al., 2009). The length of the ITI varied from 2 to 16 s, with a mean of 6.625 s. Both the length of the ITI and the order of the varying ITI were determined using Optseq2, a tool designed for optimal stimulus presentation for fMRI designs (http://surfer.nmr.mgh.harvard.edu/optseq/). The same trial randomization was used for all subjects in order to take advantage of the Optseq2 optimizations.

#### FMRI acquisition and behavioral data collection

Subjects were scanned using a Siemens Skyra 3-Tesla scanner equipped with a 16 channel phased array head coil. Stimuli were projected onto a visual display in the magnet's bore and viewed by the subject through a mirror above his or her eyes. Subjects responded with a handheld response box, and the experiment onset was synchronized with scanner activity through a trigger system.

Prior to functional data collection, a T1-weighted 3D structural volume (Siemens MPRAGE) was collected from each subject. Functional series were collected during participation in the two memory tasks, JOR and JOP. Functional T2*-weighted images were collected using an echoplanar image (EPI) sequence. Thirty-four 3 mm oblique axial slices with 2.97 mm by 2.97 mm in-plane resolution were acquired in an interleaved fashion (*TR* = 2000 ms, *TE* = 34 ms, flip angle 71°). A total of 6 functional runs were collected with three runs for each task. Each run was roughly 7 min and included 210 whole-brain acquisitions (i.e., TRs).

#### Run and trial sequences

Each participant completed 20 practice trials prior to functional data collection. Each of the 6 functional runs was composed of 32 task trials, and subjects completed 96 total trials of each task. The order of the tasks was counterbalanced, so that half of the subjects began the session with JOR and the other half began with JOP. Subjects completed 3 runs of one task, were given a brief break, and were then briefed about the change in task. Before the second task began, subjects were reminded about the nature of the new task requirements and were probed to make sure they understood the change in task-demand.

#### Image analysis and preprocessing

Preprocessing and analysis were conducted using AFNI (Cox, 1996). Data underwent the follow preprocessing steps prior to statistical analysis. Individual slice time-series were shifted to compensate for interleaved collection of slices, and both functional and structural images were re-sampled from oblique to cardinal coordinates. A despiking procedure was used to reduce the impact of artifactual outliers on the dataset. Both structural and functional data were aligned through a procedure that registered each of the functional volumes to the 4th volume of the first functional run using a 6-parameter affine motion-correction algorithm, and then aligned all functional acquisitions to the individual subject's high-resolution structural image. Spatial smoothing was applied to functional images with a 6-mm full-width half-maximum Gaussian kernel. Signal was also percentized using the mean value of each run so that beta weights could be interpreted as percent signal change. For group analyses, structural data were converted into a normalized template available through afni and in Talairach space.

Analyses were implemented using a general linear model (GLM) approach. Models included regressors of non-interest for the six motion parameters resulting from the motion correction step, as well as for the cubic polynomial trends in the run-wise data. To model multiple task events, separate regressors were entered for the encoding-maintenance phase (the interval from presentation of the first item through the end of the mask), retrieval phase (including 8 separate regressors for each correct serial position in each task), and for extended baseline periods associated with the ITI (specifically, the final 4 s of ITIs exceeding 10 s were modeled in order to establish an optimal estimate of baseline activity). Retrieval events were modeled using a single parameter gamma-variate function approximating the shape of the canonical hemodynamic response. Encoding-maintenance regressors were modeled using a one parameter block stimulus of duration 3.75 s that was also convolved with a gamma-variate model. The ITI was modeled as a block period of 4 s with no convolution.

In serial position comparisons, regressors were entered for the retrieval phase and ITI periods exceeding 10 s. Main analyses included 8 separate retrieval period regressors based on the serial position of the correct response and divided by task (i.e., following method 1). Follow-up analyses based on method 2 (i.e., using retrieval period regressors that considered both items in the probe) were also conducted.

Individual subject data were analyzed with a subject-specific fixed-effects model, and contrasts of interest were submitted to a second-level random effects group analysis. The criterion for significant clusters was calculated using AFNI's 3dclustsim program with 10,000 Monte Carlo simulations to establish family-wise error (FWE) rates. Correction to a FWE rate of 0.05 required an uncorrected voxel-wise threshold of *p* < 0.001, with a cluster size of 17 contiguous voxels. For both the encoding and retrieval phase data, this correction resulted in several very large clusters containing multiple local maxima. In these instances, the voxel-wise statistical threshold was extended upward to more stringent levels (*p* < 0.001, *p* < 0.0001, *p* < 0.00001) so that more specific peak loci within these large clusters could be identified.

### Results

#### Behavioral results

Overall accuracy was quite high (JOR: *M* = 0.92, *SD* = 0.06; JOP: *M* = 0.90, *SD* = 0.06), and reaction time for JOR and JOP were *M* = 1608.77, *SD* = 229.43, and *M* = 1491.95, *SD* = 257.43, respectively. Behavioral analyses were consistent with those performed in Experiment 1. Serial position analyses focused on reaction time in order to focus on how differences in retrieval speed may be considered differences in memory state, and due high accuracy which corresponded to very few errors per condition.

***Method 1. primacy and recency assigned according to the position of the correct response.*** A repeated measures ANOVA examining the effect of task and serial position on reaction time revealed significant interactions between task and serial position of the correct response [*F*_(3, 81)_ = 5.271, *p* = 0.006, η^2^_*p*_ = 0.387]. Again, primacy and recency effects were calculated using method 1 and the resulting indices were compared across JOR and JOP to test whether the items with the quickest retrieval speed (and arguably the most heightened memory state) were consistent across task. An ANOVA with task (JOR, JOP) and effect-type (primacy, recency) as within-subjects independent variables revealed that the main effect of task was not significant [*F*_(1, 27)_ = 0.02, *p* = 0.90, η^2^_*p*_ = 0.00], but the main effect of effect-type was significant, and showed overall larger primacy effects than recency effects [*F*_(1, 27)_ = 32.33, *p* < 0.005, η^2^_*p*_ = 0.55]. Similar to Experiment 1 there was a significant task by serial position interaction [*F*_(1, 27)_ = 9.86, *p* < 0.005, η^2^_*p*_ = 0.27]. This interaction was driven by a comparatively larger primacy effect in JOP than JOR [*t*_(27)_ = 2.19, *p* = 0.04], and larger recency effect in JOR than JOP [*t*_(27)_ = 2.53, *p* = 0.02]. Therefore, like Experiment 1 the magnitude of serial position effects was task-dependent, indicating that the size of the retrieval advantage for primacy or recency items shifted with task instructions.

***Method 2. primacy and recency assigned according to whether the first or last item was included in the probe.*** Primacy and recency effects were calculated using method 2 and the results were entered into a task (JOR, JOP) by effect type (primacy, recency) ANOVA. There was no main effect of task [*F*_(1, 27)_ = 1.05, *p* = 0.31, η^2^_*p*_ = 0.04], a larger primacy effect than recency effect [*F*_(1, 27)_ = 38.83, *p* < 0.005, η^2^_*p*_ = 0.59], and a task by effect type interaction [*F*_(1, 27)_ = 9.91, *p* = 0.006, η^2^_*p*_ = 0.25]. Paired comparisons revealed a similarly sized recency effect across tasks [*t*_(27)_ = 1.67, *p* = 0.11], but a stronger primacy effect in JOP than JOR [*t*_(27)_ = 2.80, *p* = 0.01].

#### Imaging results

Separate analyses probed activation patterns during two segments of the task: (1) the encoding-maintenance period, which included item presentation and the mask between encoding and retrieval, and (2) the retrieval period, based on the moment at which the test probe was shown. For each task (JOR, JOP), contrasts compared activation during each of these periods to baseline activation during the ITI. Tables listing the outcomes of the full set of imaging contrasts are provided in the Supplemental Materials.

The encoding-maintenance period of the both JOR and JOP were marked by significant positive activations (when compared to the ITI) in several regions, including: bilateral premotor cortices, extending through lateral portions of BA 6 and 4, the left supplementary motor area (SMA) including medial regions of BA 6 extending down to BA 32 in the dorsal anterior cingulate cortex, and bilateral activations in the posterior parietal lobe that extended through the superior and inferior parietal lobes (BA 7 and 40) via the intraparietal sulcus. Areas showing higher activation for the ITI relative to the encoding-maintenance period (i.e., deactivation) also included regions in the posterior parietal lobe (BA 40, 7), located superior to the regions of activation, and deactivation was also seen in medial regions of the right lateral prefrontal cortex (BA 8). Nearly identical patterns of activation were found for encoding-maintenance across tasks, and in fact, not a single cluster of activation reached significance in a direct comparison of encoding-maintenance activity during JOR vs. JOP.

The retrieval period was also investigated in comparison to the ITI, separately for each task, and between tasks. The retrieval period of both JOR and JOP yielded increased activations in several areas when compared to the ITI. These areas included bilateral regions of the premotor cortex (BA 4, 6), the right prefrontal cortex (BA 9), and the posterior portion of the left cingulate gyrus (BA 23). A few regions also showed higher activity levels during the ITI than retrieval, including the MTL, the inferior frontal gyrus (in BA 47), and posterior parietal lobe (BA 7, 40). Again, activations were highly consistent across the JOR and JOP tasks, and there were no clusters of activation that varied significantly in a direct comparison of retrieval-related activity associated with the two tasks. Notably, overall activation patterns in both the encoding-maintenance and retrieval periods were consistent across tasks, and also consistent with a network of regions in the frontal and parietal lobes observed in numerous other fMRI examinations of WM (Wager and Smith, 2003; Owen et al., 2005).

***Method 1. primacy and recency assigned according to the position of the correct response.*** The neural correlates of the behavioral primacy and recency effects were examined by analysis of trial activity dependent on the serial position of the correct probe. In order to maintain consistency with Öztekin et al. (2009), analyses examined activation patterns during the retrieval period of the task. Recency effects compared fMRI signal on recency item trials and middle item trials, while primacy effects compared fMRI signal on primacy item trials and middle items trials, and these trials were defined identically to Experiment 1. FMRI contrasts exploring recency effects showed no above threshold differences between recent and middle item trials in either the JOR or JOP task. Additional analyses were conducted in order to further probe recency effects. These included comparison of probes involving the recency item to probes involving only the item immediately preceding the final item (i.e., item 3 in JOP and item 4 in JOR), and comparing recency item probes to all other types of probe. These analyses also failed to show above threshold clusters of activation. Therefore, in this pool of subjects, there were not any distinct neural correlates of trials probing the most recent item when compared to trials probing other items.

**Figure 4:**
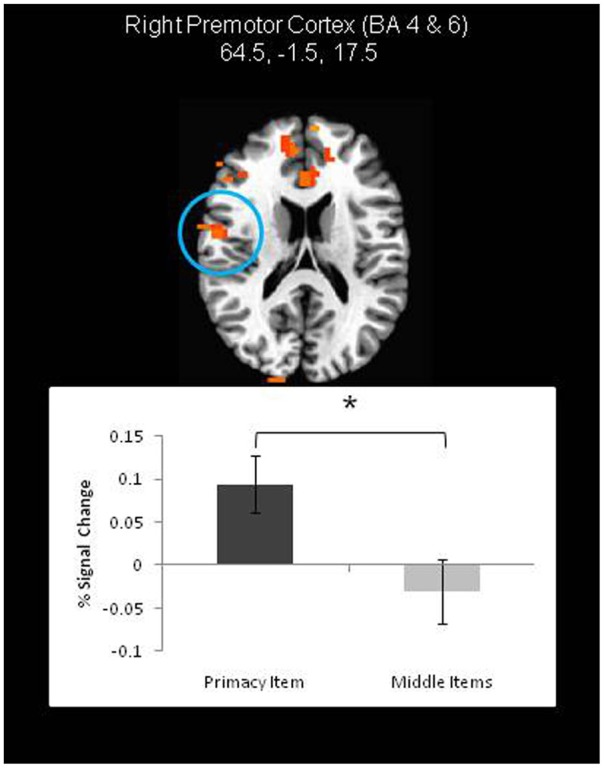
**A frontal lobe region exhibiting higher activation during primacy item trials than recency item trials during the retrieval period of a JOR task**. In other words, a neural correlate of the behavior primacy effect. ^*^ denotes significance at *p* < 0.05.

On the other hand, neural correlates of the primacy effect were found in both the JOR and JOP tasks (Tables 1, 2). In JOR, regions in the right premotor cortex (extending through frontal areas associated with BA 4 and 6), and right occipital/fusiform gyri (BA 18, 19, 37) displayed higher activation for primacy trials than middle trials. Figure 4 depicts a region in the right premotor cortex exhibiting differential activation across primacy and middle trials. In addition to these regions, a few regions showed significantly different levels of deactivation (i.e., activation below baseline), including the left anterior cingulate (BA 32, BA 24) and the right inferior frontal gyrus (with a peak activation in BA 47 and extending through portions of BA 45). This investigation of primacy effects in a JOR task did not reveal any above threshold clusters with higher activation for middle item probes relative to primacy item probes.

**Table 1:** **Regions identified during serial position contrasts in the JOR task**.

**Location**	**BA**	***p*-value**	***X***	***Y***	***Z***
**2 > 3 and 4**					
*Left superior frontal gyrus*	*9*	*0.001*	−*13.5*	*58.5*	32.5
*Right inferior frontal gyrus*	*45, 46, 47*	*0.001*	*49.5*	*34.5*	−*6.5*
*Left anterior cingulate*	*32, 24*	*0.001*	−*1.5*	*34.5*	*11.5*
*Right middle temporal gyrus*		0.001	49.5	10.5	−30.5
*Right cuneus*	*18*	*0.001*	*10.5*	−*94.5*	*20.5*
Right precentral gyrus	6, 4	0.001	64.5	−1.5	17.5
Right fusiform gyrus	37	0.001	46.5	−52.5	−15.5
**2 < 3 and 4**					
None					
**2 > 5**					
*Right fusiform gyrus*	19	*0.001*	*40.5*	−*67.5*	−*15.5*
**2 < 5**					
None					

Italicized lines denote clusters of deactivation that are significantly below baseline.

**Table 2:** **Regions identified during serial position contrasts from the JOP task**.

**Location**	**BA**	***p*-value**	***X***	***Y***	***Z***
**1 > 2 and 3**					
*Left middle frontal gyrus*	*6, 8*	*0.001*	−*28.5*	*22.5*	*53.5*
*Left angular gyrus*	*39*	*0.001*	−*49.5*	−*70.5*	*35.5*
*Right superior frontal gyrus*	*8*	*0.001*	*16.5*	*46.5*	*41.5*
*Right inferior frontal gyrus*	45, 46	*0.001*	*55.5*	*31.5*	*5.5*
*Right middle frontal gyrus*	11	*0.001*	*37.5*	*34.5*	−*12.5*
**1 < 2 and 3**					
Left precentral gyrus	6	0.001	−49.5	−1.5	44.5
Left medial frontal gyrus	6, 32	0.001	−1.5	7.5	47.5
Left inferior occipital gyrus	19	0.001	−46.5	−79.5	−3.5
Right thalamus		0.001	10.5	−4.5	11.5
Medial cerebellum		0.001	4.5	−49.5	−24.5
Left middle frontal gyrus	46	0.001	−43.5	19.5	26.5
**4 > 1**					
None					
**1 < 4**					
Right cuneus	17, 18	0.001	10.5	−79.5	8.5

Italicized lines denote clusters of deactivation that are significantly below baseline.

**Figure 5:**
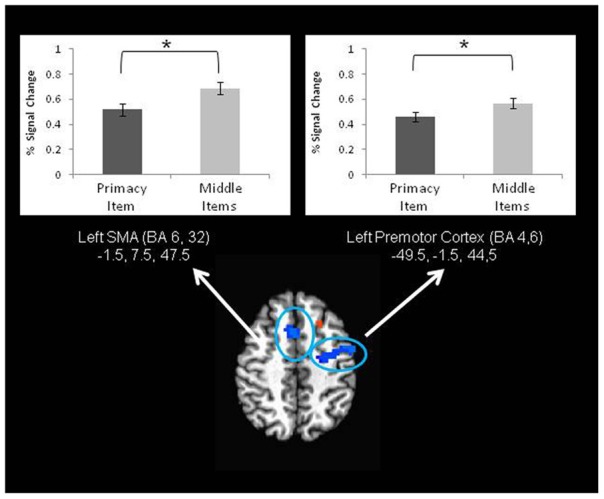
**Two frontal lobe regions exhibiting higher activation during middle item trials than primacy item trials during the retrieval period of a JOP task**. This represents a neural correlate of the behavioral primacy effect in a JOP task. ^*^ denotes significance at *p* < 0.05.

For JOP there were also significant differences in brain activation that corresponded to the behavioral primacy effect, but the pattern of results was different from that obtained with the JOR task. With JOP, significantly lower activation for primacy item trials than for middle items trials was found in several areas, including the left premotor cortex (lateral portions of BA 6 and BA 4), SMA (medial portions of BA 6 and BA 32), and dorsolateral prefrontal cortex (BA 46) (Figure 5).

Contrasts also compared the retrieval period of primacy and recency item probes to each other. The purpose of these contrasts was two-fold: to compare the neural mechanisms of retrieval of the earliest and latest items, and also to “complete the set” of comparisons between the three trial types (primacy item trials, middle item trials, and recency item trials). In each case, regions were very consistent between the primacy and recency probe trials, and only areas of the right occipital and fusiform gyrus (BA 17, 18) varied between primacy and recency item probes. In JOR, activity was higher for the primacy item probe than the recency item probe, while in JOP it was higher for the recency item probe than the primacy item probe.

In order to more formally test whether task-demands affect the neural signature of serial position effects, ANOVAs comparing primacy and recency effects observed in each task were conducted. The first ANOVA included middle and recency trials (JOR: items 3, 4, 5; JOP: items 2, 3, 4) across task (JOR, JOP), while the second examined primacy and middle trials (JOR: items 2, 3, 4; JOP; 1, 2, 3) across task (JOR, JOP). The results of interest regarded the task by effect-type interaction.

A single inferior frontal region (BA 9/44) showed a significant task by recency effect interaction. The source of this interaction was explored through comparison of fMRI signal change (against baseline) in each of the serial positions of interest. However, contrasts revealed that neither JOR nor JOP showed a difference between the recency trial and the middle trials [JOR: *F*_(1, 27)_ = 2.32, *p* = 0.14, η^2^_*p*_ = 0.08: JOP: *F*_(1, 27)_ = 1.78, *p* = 0.20, η^2^_*p*_ = 0.06]. Instead this task by serial position interaction was driven by a difference between performance on middle trials that was found in JOR [*F*_(1, 27)_ = 20.60, *p* < 0.005, η^2^_*p*_ = 0.43] and not in JOP [*F*_(1, 27)_ = 0.12, *p* = 0.72, η^2^_*p*_ = 0.01]. In other words this difference was not driven by a difference in the neural response for retrieval of recency items.

**Figure 6:**
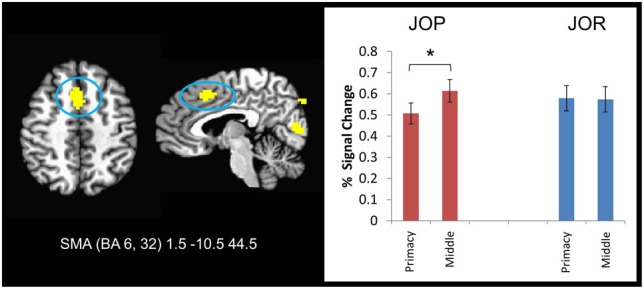
**A frontal lobe region showing an interaction between task and serial position on activation during the retrieval period of JOR and JOP**. During the JOP task, individuals showed a higher level of activation during middle item trials than during the primacy item trials. No such difference emerged during the JOP task. ^*^ denotes significance at *p* < 0.05.

Additional support for the task-dependent nature of the neural correlates of primacy was provided by the serial position by task ANOVA. Specifically, 9 different regions showed a task by effect-type interaction, including a medial frontal cluster extending through the SMA and the dorsal anterior cingulate cortex (BA 6, 8, 32), bilateral regions in the inferior frontal gyri in BA 46 that extended through BA 9, and a region in the right middle frontal gyrus (extending though BA 6 and 9). Investigation of the source of the task by serial position interaction corroborates the within task findings regarding primacy. In 7 of the 9 significant clusters of activation, JOP showed less activity associated with retrieval during primacy item probe trials than retrieval during middle item probe trials. In JOR, one of two patterns emerged, no difference between primacy and middle trials or stronger activation during primacy item trials than middle item trials. This pattern is illustrated in a region of the medial frontal cortex (SMA, Figure 6), a region overlapping with one implicated in the within task primacy effect analyses. A significant neural primacy effect was found in JOP [*F*_(1, 27)_ = 15.28, *p* = 0.001, η^2^_*p*_ = 0.26], but no such difference was found in JOR [*F*_(1, 27)_ = 0.02, *p* = 0.899, η^2^_*p*_ = 0.00].

***Follow-up analyses in the MTL.*** The primary serial position comparisons in the present paper did not reveal differences in the MTL, and analyses of both the encoding-maintenance and retrieval periods compared to the ITI revealed lower activation during encoding-maintenance and retrieval than at rest. However, as we mentioned above, prior studies have found MTL differences using a variety of specific serial position contrasts. For example, (Talmi et al., 2005) compared probes involving the first two items (in a 12 item list) to probes involving the last two items. To mirror this analysis on our shorter list, we compared probes involving the first two and last two possible correct items in both JOR (positions 2 and 3 against 4 and 5) and JOP (positions 1 and 2 against 3 and 4). Using a lowered uncorrected threshold of *p* < 0.005, two small clusters showed relatively higher activation in the left MTL for trials with earlier correct answer than trials with later correct answers. However, these clusters were small (6 and 9 voxels), and comparison to the rest period revealed that activity in both groups of items was lower than activity at rest. Comparisons based on JOP did not reveal any relevant clusters. As these results were task specific, and the clusters found in JOR were only sub-threshold, we are hesitant to interpret these differences as strong indicators of differences in retrieval operations.

***Method 2. primacy and recency assigned according to whether the 1st or last items are included in the probe.*** In a set of follow-up analyses mirroring the behavioral results, trials were divided according to the serial position of both probes. Tables reporting these findings can be found in the Supplemental Materials. Consistent with method 1, in JOR and in JOP there were no above threshold clusters of activation that differed between recency trials and middle trials. In the case of primacy in JOP, a set of frontal regions (including regions of BA 6, and BA 46) showed lower levels of activation in primacy item trials than middle item trials. No such pattern was revealed in JOR.

### Discussion

Consistent with the findings from Experiment 1, the comparative size of the behavioral primacy and recency effects shifted with the demands of the task. Once again, both methods of calculating primacy and recency effects yielded a larger recency effect in JOR than JOP, and a larger primacy effect in JOP than JOR. Accordingly, both experiments provide evidence that the presence of primacy and recency effects does not signal some immutable trait of WM, but rather, indicates a task-dependent emphasis on different items in the list.

Imaging analysis of the encoding-maintenance and retrieval periods of JOR and JOP showed very consistent patterns of activation across tasks, and only when specific serial positions were considered did differences emerge. Examination of the encoding-maintenance and retrieval periods of JOR and JOP showed bilateral frontal (BA 4, 6, 32), and parietal lobe (BA 7, 40) activations consistent with numerous prior investigations of WM (Wager and Smith, 2003; Owen et al., 2005). A newer development to the fMRI literature on WM regards the role of the MTL in short-term WM tasks (Ranganath and D'Esposito, 2001; Cabeza et al., 2002; Ranganath et al., 2004; Chein et al., 2011). The present study did not find positive activation of the MTL, and in fact, found significantly reduced activation in bilateral regions of the MTL relative to rest.

Central to the present work was the aim of characterizing the neural correlates of primacy and recency effects. Imaging contrasts failed to identify any brain regions where activity specifically varied in association with retrieval of an item in the recency position (e.g., a recency effect). This finding is consistent with the small behavioral recency effect reported in this cohort of subjects, but is inconsistent with the prior literature on the neural markers of recency effects (Öztekin et al., 2009).

In contrast to a null result with respect to recency, contrasts revealed differences in fMRI signal that could be linked to the primacy effect. In the case of JOR, *higher* activation during primacy item trials than middle item trials was found in regions of the right premotor cortex (BA 6 and 4) and the right fusiform gyrus. Notably, in JOP, fMRI signal was *lower* for primacy item trials than middle item trials in regions including the left premotor cortex (BA 4, 6) and SMA (BA 6, 32). Consistent with this apparent task-dependent pattern, a direct analysis of the interaction between neural signatures of the primacy effect and task (JOR and JOP) revealed an interaction in a group of regions including the SMA (BA 6, 32), and right inferior frontal gyri (extending from BA 46 to BA 6 and 9). Further exploration of the SMA (and several other areas) showed that this interaction resulted from weaker activation for primacy item trials than middle item trials in JOP, but no such difference for JOR.

In sum, Experiment 2 yielded two main findings. First, like Experiment 1, there was an effect-type by task interaction showing a larger advantage of primacy in JOP and larger advantage of recency in JOR. Second, FMRI contrasts demonstrated task specific neural correlates of the primacy effect, which differed between JOR and JOP.

## General discussion

The present work examined serial position effects and evidence supporting the claim that the FOA, the most immediate state of WM, is limited to a single item. Evidence for this limited FOA is drawn from observation of recency effects (McElree and Dosher, 1993; McElree, 2006; Öztekin et al., 2009). We investigated both recency and primacy effects and asked whether the qualities of recency and primacy effects are consistent across manipulation of assessment type, and whether recency effects and/or primacy effects should be interpreted as indicative of representation in the FOA. We found that the comparative size of primacy and recency effects shifted with the manipulation of task-demand. Both experiments revealed a larger recency effect in JOR than JOP and larger primacy effect in JOP than JOR. This finding was consistent across two different analysis strategies, one which defined primacy and recency trials according to the serial position of the correct item, and another which defined primacy and recency trials according to whether the first or last item was contained in the probe as either the correct or incorrect item. The task-dependence of primacy and recency effects provides a clear indication that these phenomena alone cannot be relied upon as markers for fixed properties of WM.

Imaging analyses of the encoding-maintenance and retrieval periods of JOR and JOP showed activation patterns consistent with a network of frontal and parietal regions that is commonly implicated in maintenance, monitoring and updating of information in WM (Miller and Cohen, 2001; O'Reilly and Frank, 2006; Chein et al., 2011). Moreover, consistent with previous studies (Rypma and D'Esposito, 1999; Kruggel et al., 2000; Chein and Fiez, 2001), several of these frontal cortex regions were activated at the encoding-maintenance phase and also the retrieval phase of the task.

The present study did not reveal increased activation of the MTL during either encoding-maintenance or retrieval (when compared to activity during the ITI). This is inconsistent with recent studies examining both JOR (Öztekin et al., 2009) and item recognition tasks (Nee and Jonides, 2008, 2011; Öztekin et al., 2010). Importantly, investigation of the role of the MTL in WM is rather new to the study of WM and its neural underpinnings. Many of the demonstrations of the role of the MTL in WM occurred in studies where items were unfamiliar, complex, or involved relational processing (Cabeza et al., 2002; Ranganath et al., 2004; Olson et al., 2006). For example, two recent examinations showed MTL activation during performance on complex span tasks, which interweave each item in a list with an unrelated processing task (Chein et al., 2011; Faraco et al., 2011).

Together, the absence of MTL activation in this task, and the presence of MTL activation in studies where items were unfamiliar, complex, or involved relational processing suggests a possible interpretation of the role of the MTL in WM. Perhaps the MTL is recruited when other mechanisms, such as those that support active maintenance, are exhausted. It follows that the tasks used here may not have adequately targeted the MTL because resources used to maintain and rehearse items were not exhausted. High levels of accuracy in these subjects indicate that they were able to actively rehearse or maintain the items. Further research is necessary to elucidate the precise role of the MTL in WM performance and the specific conditions when the MTL is, and is not, engaged in WM.

### Neural correlates of recency and primacy

The most surprising finding in the present study was the lack of a neural signature for the behavioral recency effect in either task. This is in stark contrast to earlier studies that reported a neural signature of the recency effect (e.g., Öztekin et al., 2009, 2010). One conclusion that can be drawn from the absence of a neural recency effect is that the FOA is not necessarily committed to the last item in a list, and we favor this conclusion.

Neural correlates of the behavioral primacy effect were observed in both JOR and JOP, a pattern that is broadly consistent with the finding that during an item recognition task, recognition of early items in a list reveals a different pattern of activation than recognition of later items in a list (see Talmi et al., 2005). In JOR, a region in the right premotor cortex (BA 4 and 6) showed greater activation during the retrieval period in primacy item trials than middle item trials; whereas, in JOP, regions of the left premotor cortex and SMA showed less activation during retrieval in primacy item trials than middle item trials. Additional analyses demonstrated a task by effect-type interaction in regions such as the SMA (BA 6, 8, 32) and right and left inferior frontal gyrus in BA 46. Several of these regions, including the SMA and left premotor cortex, are strongly implicated in phonological rehearsal of items (Awh et al., 1996; Smith and Jonides, 1998). It follows that one explanation of the primacy effect links it to variation in rehearsal processes at the time of retrieval. In JOR, activity was higher for the primacy item trials than middle item trials, and in JOP, activity was lower for primacy item trials than middle item trials. Finding either the pattern associated with JOR or the pattern associated with JOP would lead to a directional prediction about the relationship of rehearsal processes to the primacy effect. However, together the findings from JOR and JOP tell a more complex story about the neural correlates of primacy.

At least two accounts, both somewhat speculative, could explain these task-related differences. First, the JOR and JOP tasks might engage rehearsal processes differently, leading to differences in the source of the behavioral primacy effect. For example, in JOP, subjects might direct more rehearsal efforts toward earlier items in the list during encoding. If the earliest item is well rehearsed and encoded, it may require less re-checking via rehearsal at test than items that were not as well rehearsed. In contrast, the objective in a JOR task might direct subjects away from rehearsing earlier items, and a primacy effect could emerge from stronger rehearsal processes at test. In a different but related explanation, the neural correlates of primacy found in JOP might be specific to the very first item in a list, which is not the primacy item in JOR (because the first item cannot be the most recent item). This interpretation indicates a one item capacity for this sort of primacy effect. If this were the case, then the primacy effect in JOR would necessarily arise from a separate mechanism. Again, this account could be linked to stronger rehearsal processes for primacy item trials (in JOR) and less a necessity of rehearsal processes for primacy item trials (in JOP). The present pattern of findings does not distinguish between explanations. However, the novel findings above suggest that primacy effects in WM could be tied to the strength of active-maintenance processes during the retrieval period of WM tasks. Moreover, the task dependent effects found here suggest that focusing on a single task could lead to a simpler but potentially misleading story.

Notably, the JOR task was designed to “eliminate or minimize engagement in maintenance rehearsal operations, so that retrieval specific differences in neural activation across tasks and [serial position] could be examined without confounding effects of encoding and maintenance operations” (Öztekin et al., 2009, p. 583). Cowan (2001) also suggests that rehearsal processes should be limited during measurement of the FOA. Although the JOR task used in the present study was nearly identical to the one used by Öztekin et al. (2009), and the JOP task used identical timing procedures, the pattern of brain activation suggests that rehearsal operations were present. It follows that the presence of supplementary rehearsal processes may have obscured measurement of a single item FOA tied to the final item, and led to results that are inconsistent with Öztekin et al. (2009) findings. However, a central goal of the present work was to illuminate the architecture of WM states, and rehearsal processes (and their neural correlates) are an essential feature of WM performance (Baddeley et al., 1998; Davachi et al., 2001; Wager and Smith, 2003; Camos et al., 2011). The present findings imply that rehearsal processes can be present even in a task designed to limit them, and we contend that the presence of rehearsal in the present study need not invalidate measurement of the underlying structure of WM.

### Comparison of past and present methodologies

While the present experiment was meant to replicate and extend Öztekin et al. (2009), a few differences in methodology between these two studies should be noted. Importantly, the JOR task in the present study was nearly identical to the task from Öztekin et al. (2009). The one exception was that 16 rather than 20 consonants were sampled in the present study. The overall experimental sessions did differed in two ways. In Öztekin et al. (2009) subjects practiced the task for 45 min (compared to roughly 10 min in the present study). Moreover, in many of the earlier behavioral studies of JOR, even higher levels of practice were involved (Muter, 1979; Hacker, 1980; McElree and Dosher, 1993). For example, in Experiment 1 of McElree and Dosher (1993), subjects completed 20, 75 min sessions, and in Hacker (1980) subjects completed 75, 5 min sessions. Importantly, larger amounts of practice may have led to different performance levels and/or approaches to the task. For example, extensive practice with a task may prompt subjects to extend less effort to sustain items in a heightened state at encoding. Instead subjects could learn to rely more on retrieving some or the entire list of items during the retrieval period.

Another key difference between the present paper and Öztekin et al. (2009) was the intermixing of different trial types. In the present experiment, trial type was always blocked. In contrast, Öztekin et al. (2009) intermixed trials of item recognition and JOR, and subjects did not know the requirement of the task until the retrieval probe was presented. Not knowing whether order or identity would be required at test may have led to a different task approach (or differential emphasis on WM processes). Importantly, Berryhill et al. (2011) found that, when two tasks were administered in different blocks, differing strategies could be allocated to each one, whereas when the task varied, subjects reverted to a single strategy. In the present study, differences in the level of practice and the details of the session may have contributed to the differences in findings reported here and in past studies. If so, this is consistent with the suggestion that neural and behavioral signatures of WM are tied to very specific properties of a memory task.

### Implications for the structure of WM

What do these findings contribute to our understanding of the structure of WM and the FOA? This question can be broken into two sub-questions: Is the primacy effect seen here indicative of the FOA? And, how do these findings support or refute theoretical accounts of the FOA? Distinct neural and behavioral characteristics of a primacy effect were reported in a JOR and a JOP task. If we define the FOA as the most immediate state of WM where item(s) are in a heightened state of activation (and can be retrieved faster than items outside of this state), then the primacy effect observed here is indeed indicative of a FOA, which is capable of maintaining multiple items, including those from the beginning of the stimulus lists.

The present results contribute to discussion of three competing accounts of the FOA, McElree's single item FOA (McElree and Dosher, 1989, 1993; McElree, 2006), Cowan's multiple item FOA (Cowan, 1995, 1999, 2001), and Oberauer's three level model of memory (Oberauer, 2002; Oberauer and Lange, 2009). Importantly, both Oberauer's and McElree's models suggest a one item FOA limited to the final item in a list. Therefore, the evidence of neural correlates of primacy only is inconsistent with the assumptions of these accounts. The behavioral recency effect could be seen as indicative of the FOA, but only if such an account could reconcile the lack of a neural signature of recency effects seen here.

If these results do not support a single item FOA, do they instead support Cowan's account of a multiple item FOA (Cowan, 1995, 1999, 2001)? Several aspects of the findings do seem in line with the notion of a multiple item FOA. Subjects responded with high accuracy to trials presenting five different items in an immediate memory task. Moreover, the fMRI data show activation of frontal and parietal regions associated with WM task performance (Wager and Smith, 2003; Owen et al., 2005) and not regions like the MTL which are more typically associated with retrieval from longer term memory (Hermann et al., 1996; Schacter and Wagner, 1999; Golby et al., 2001). Therefore, one interpretation of these results is that several of the items in the JOR and JOP tasks were in the FOA, a heightened state of awareness separate from long-term memory. Notably, this argument involves dismissal of recency or primacy effects as necessary markers of the FOA. Without dismissal of serial position effects as a marker of the FOA there is preliminary evidence from Experiment 1 for a multiple item FOA. JOR trials containing the first and last items were completed faster than primacy trials (containing the first but not the last item) or recency trials (containing the last but not the first item). This may suggest that both the first and the last item are in the FOA. However, in the present experiment there were very few (only 8) of this trial type, and so conclusions based on this observation should be met with caution. On the other hand, this study might point not to a multiple item FOA, but an FOA that can be flexibly allocated (no matter its size) according to task parameters. In the present study, the JOP task may have encouraged individuals to maintain only the primacy item in a heightened cognitive state (a pattern not revealed in the extant literature). As Cowan's model presupposes not only a multiple item FOA, but also the ability to flexibly allocate this heightened state of awareness (Cowan, 1995, 1999, 2001), we argue that the present findings – while complex– are more easily accommodated by Cowan's notion of the FOA.

In sum, the results above do not suggest a distinct cognitive status (e.g., the FOA) tied to the last item in a list. Instead these results suggest that both primacy and recency effects are task-dependent performance metrics. Consequently, as the data here shows a more complex outcome than did prior studies (Öztekin et al., 2009, 2010), we argue that it is important to proceed with caution when interpreting recency effects as a stable, immutable mark of the architecture of WM. Instead, these results are consistent that the notion that individuals can flexibly allocate attentional resources according to task demands, and that this allocation is not tied to a particular item in a list.

### Conflict of interest statement

The authors declare that the research was conducted in the absence of any commercial or financial relationships that could be construed as a potential conflict of interest.
