# Supplementary material for: Primacy and recency effects as indices of the focus of attention
Source: Front Hum Neurosci. 2014 Jan 24;8:6. doi: 10.3389/fnhum.2014.00006 (PMC3900765; doi:10.3389/fnhum.2014.00006)
Supplement: Supplementary file 1 [file Presentation1.PDF]

Supplemental Materials

Talaraich Tables from fMRI Analyses in Experiment 2.

Table S1. Primacy Effect: Task (JOR, JOP) by serial position (middle and primacy item trials) Interaction using Method 1.

Location	BA	p-value	X	Y	Z
Task by Primacy Effect Interaction (early item vs middle)					
Left Fusiform Gyrus	18	0.001	-31.5	-88.5	-15.5
Right Inferior occipital	18, 19	0.001	34.5	-85.5	-18.5
Right Medial Frontal	6,32	0.001	1.5	10.5	44.5
Right Cuneus	23 30	0.001	1.5	-73.5	11.5
Right Cuneus	19	0.001	4.5	-85.5	38.5
Left Inferior Frontal Gyrus	*9, 46*	0.001	-37.5	4.5	32.5
Right Middle Frontal Gyrus	9	0.001	34.5	31.5	26.5
Right Inferior Frontal Gyrus	6,	0.001	43.5	7.5	29.5
left Caudate		0.001	-4.5	13.5	5.5

Table S2. Recency Effect: Task (JOR, JOP) by serial position (middle and recency item trials) Interaction using Method 1.

Location	BA	p-value	X	Y	Z
Task by Recency Effect Interaction (Late item vs middle)					
Inferior Frontal Gyrus	9	0.001	-37.5	4.5	32.5

Table S3. Primacy Effect in JOP. Conditions defined via Method 2.

Location	BA	x	y	z
Primacy < Middle				
Left Inferior Occipital Gyrus	18	-34.5	-91.5	-9.5
Right Medial Cerebellum		40.5	-46.5	-24.5
Right Medial Cerebellum		4.5	-61.5	-0.5
Left Middle Frontal Gyrus	46	-43.5	19.5	26.5
Medial Frontal Gyrus	6	-1.5	4.5	50.5
Left Precuneus	7	-22.5	-61.5	41.5
Left Precentral Gyrus	6	-37.5	-4.5	41.5
Left Thalamus		-10.5	-10.5	-0.5
Primacy > Middle				

Left Inferior Parietal Lobe	39	-49.5	-67.5	41.5

Table S4. Judgment of Recency: Activations associated with the Encoding-Maintenance and Retrieval periods when compared to the ITI.

Judgment of Recency					
Location	BA	p-value	x	y	z
Encoding > ITI					
Right Fusiform Gyrus	18, 19, 37	0.00001	46.5	-73.5	-12.5
Left inferior Occipital Gyrus	18, 19, 37	0.00001	-34.5	-88.5	-12.5
Left Precentral Gyrus (Premotor Cortex)	4,6	0.00001	-52.5	-4.5	44.5
Right Precentral Gyrys (Premotor Cortex)	4,6	0.00001	55.5	-7.5	41.5
Left Medial Frontal Gyrus (SMA)	6, 32	0.00001	-1.5	-1.5	56.5
Left Lentiform Nucleus/Putamen		0.00001	-19.5	4.5	5.5
Right Superior Parietal Lobe	7, 40	0.00001	25.5	-61.5	44.5
Right Lentiform Nucleus/Putamen		0.00001	19.5	7.5	5.5
Left Superior Temporal Gyrus	21	0.00001	-52.5	-43.4	11.5
Left Superior Parietal Lobe	7, 40	0.0001	-25.5	-61.5	47.5
Left Thalamus		0.0001	-7.5	-16.5	11.5
Right Superior Temporal Gyrus		0.001	49.5	-37.5	8.5
Encoding < ITI					
Right Cuneus	18, 19, 37	0.0001	1.5	-82.5	35.5
Left Insula	13	0.0001	-37.5	-16.5	2.5
Right Superior Parietal Lobe (PPC)	7, 40	0.001	16.6	-55.5	62.5
Right Insula	13	0.0001	40.5	-13.5	2.5
Right Inferior Parietal Lobe (PPC)	7, 40	0.0001	61.5	-37.5	41.5
Right Paracentral Lobule	31	0.001	1.5	-10.5	44.5
Left Inferior Parietal Lobe (PPC)	7, 40	0.0001	-61.5	-34.5	29.5
Right Superior Frontal Gyrus	8	0.001	1.5	19.5	50.5
Left Precunues	7, 40	0.0001	-1.5	-55.5	32.5
Left Middle Frontal Gyrus		0.001	-34.5	1.5	56.5
Left Cuneus	18, 19, 37	0.0001	-1.5	-85.5	32.5
Retrieval > ITI					
Right Fusiform Gyrus	18, 19, 37	0.00001	46.5	-73.5	-12.5
Left Cingulate Gyrus	23	0.0001	-1.5	-28.5	26.8
Right Precentral Gyrys (Premotor Cortex)	6	0.00001	37.5	-7.5	59.5
Right Thalamus		0.00001	19.5	-28.5	0.5
Left Superior Frontal gyus	6	0.00001	-1.5	4.5	50.5
Right inferior Frontal Gyrus	13	0.00001	31.5	22.5	8.5
Right Superior Frontal Gyrus	9	0.00001	37.5	43.5	32.5
Left Cuneus		0.00001	-7.5	-73.5	8.5
Retrieval < ITI					

Left Superior Frontal Gyrus		0.00001	-10.5	-61.5	32.5
Left Inferior Frontal Gyrus	47	0.00001	-49.5	31.5	-3.5
Left Angular Gyrus	39	0.00001	-49.5	-67.5	32.5
Right Inferior Parietal Lobe		0.001	46.5	-70.5	41.5
Right Caudate		0.00001	-19.5	40.5	14.5
Right Cerebellum		0.001	25.5	-82.5	-27.5
Right Parahippocampal Gyrus	28	0.00001	-22.5	19.5	-12.5
Left Cingulate Gyrus	31	0.00001	-1.5	-49.5	26.5
Right Inferior Frontal Gyrus	47	0.00001	-49.5	-31.5	-6.5
Right Inferior Parietal Lobe (PPC)	7, 40	0.00001	-55.5	58.5	38.5
Right Insula	13	0.00001	40.5	-16.5	2.5
Left Parahippocampal	28	0.00001	-22.5	-19.5	-9.5

Table S5. **Judgement of Primacy:** Activations associated with the Encoding-Maintenance and Retrieval periods when compared to the ITI.

Location	BA	p-value	X	Y	Z
Encoding > ITI					
Right Fusiform Gyrus	18, 19, 37	0.00001	46.5	73.5	12.5
Left inferior Occipital Gyrus	18, 19, 37	0.00001	-34.5	-88.5	12.5
Left Precentral Gyrus	4,6	0.00001	-49.5	-4.5	50.5
Right Precentral Gyrys	4,6	0.00001	46.5	-7.5	53.5
Left Medial Frontal Gyrus	4,6	0.00001	-1.5	-4.5	59.5
Right Superior Parietal Lobe	7, 40	0.00001	25.5	-61.5	44.5
Left Lentiform Nucleus/Putamen		0.00001	-19.5	4.5	8.5
Left Superior Parietal Lobe	7, 40	0.00001	-25.5	-61.5	47.5
Left Thalamus		0.0001	-10.5	-16.5	8.5
Right Lentiform Nucleus/Putamen		0.00001	-19.5	7.5	5.5
Left Superior Temporal Lobe	21	0.00001	-52.5	-43.5	11.5
Encoding < ITI					
Left Lingual Gyrus	18	0.00001	-4.5	-67.5	2.5
Left Postcentral Gyrus		0.001	-16.5	-55.5	65.5
Right Insula	13	0.00001	37.5	-16.5	2.5
Right Supramarginal Gyrus	7, 40	0.0001	58.9	-55.5	35.5
Left Insula	13	0.00001	-40.5	-7.5	5.5
Right Superior Frontal Gyrus	8	0.0001	1.5	28.5	44.5
Left Parietal Lobule	40	0.001	-61.5	-34.5	29.5
Right Angular gyrus	39, 19	0.001	40.5	-76.5	32.5
Left Inferior Parietal Lobe		0.001	-43.5	-55.5	38.5
Left middle Frontal Gyrus		0.001	-43.5	-16.5	41.5
Right Inferior Frontal Gyrus	45	0.001	49.5	19.5	14.5

Left Superior Parietal Lobe	7, 40	0.00001	-16.5	-55.5	62.5
Retrieval > ITI					
Right Fusiform Gyrus	18, 19, 37	0.00001	46.5	-73.5	12.5
Left Cingulate Gyrus	23	0.00001	-1.5	-28.5	26.5
Right Superior Frontal Gyrus	9	0.00001	-34.5	49.5	29.5
Left Cuneus		0.00001	-7.5	-73.5	8.5
Right Precentral Gyrys	6	0.000001	-43.5	-4.5	53.5
Right Middle Frontal Gyrus	9	0.000001	34.5	43.5	29.5
Retrieval < ITI					
Right Superior Frontal Gyrus		0.00001	-10.5	61.5	32.5
Left Inferior Frontal Gyrus	47	0.00001	-49.5	31.5	-3.5
Left Precuneus	19	0.00001	-43.5	-70.5	41.5
Right Inferior Parietal Lobe	39	0.001	49.5	-64.5	41.5
Right Caudate		0.00001	19.5	-40.5	14.5
Right Cerebellum		0.001	25.5	-82.5	27.5
Right Cerebellum		0.001	-1.5	-40.5	39.5
Left Inferior Frontal Gyrus	47	0.0001	52.5	28.5	-0.5
Left Cingulate Gyrus	31,23	0.00001	-1.5	-49.5	26.5
Right Angular gyrus	39	0.00001	49.5	-67.5	38.5
Right Parahippocampal Gyrus		0.0001	25.5	-16.5	12.5
Right Cerebellum		0.0001	22.5	-82.5	27.5
Right Inferior Frontal Gyrus	47	0.00001	52.5	28.5	-0.5
Left Parahippocampus		0.00001	-31.5	-40.5	0.5
